# Assessment of Risk Factor for Cardiovascular Disease Using Heart Rate Variability in Postmenopausal Women: A Comparative Study between Urban and Rural Indian Women

**DOI:** 10.1155/2013/858921

**Published:** 2013-07-11

**Authors:** Nikhil Narayanaswamy, Shailaja Moodithaya, Harsha Halahalli, Amrit M. Mirajkar

**Affiliations:** ^1^K S Hegde Medical Academy, Nitte University, Mangalore 575 018, India; ^2^Department of Physiology, K S Hegde Medical Academy, Nitte University, Mangalore 575 018, India

## Abstract

Cardiovascular diseases are important causes of morbidity and mortality in postmenopausal women. A major determinant of cardiovascular health is the status of autonomic nervous system and assessment of Heart Rate Variability (HRV). Heart Rate Variability is a noninvasive and sensitive technique to evaluate cardiovascular autonomic control. Reduced HRV is an independent risk factor for the development of heart disease. This study evaluated the risk factors for cardiovascular diseases using HRV, between urban and rural Indian postmenopausal women ranging in age from 40 to 75 years. Findings of the analysis of HRV have showed that the total power which reflects overall modulation of cardiac autonomic activity (759 ± 100  versus 444 ± 65), the absolute power of high frequency which is surrogate of cardiovagal activity (247 ± 41  versus 163 ± 45), and low frequency that reflects cardiac sympathetic activity (205 ± 26  versus 127 ± 18) were significantly higher in urban women than that of their rural counterparts. Further, among the anthropometric measures, waist circumference was significantly correlated with indices of HRV. The study concludes that rural Indian women are associated with an additional risk beyond that of ageing and postmenopausal status when compared to the urban women. The higher central obesity could be the contributing factor for developing higher risk for cardiovascular disease among the rural women.

## 1. Introduction

As women age, their health is influenced by factors such as career, diet, physical activity level, the socioeconomic status, and environment [1]. These changes, together with natural menopause, process of ageing, and hormonal changes in the reproductive system, affect the well-being of women. The complex, interrelated nature of the process often makes it difficult to distinguish between the symptoms of ageing or those resulting from the loss of ovarian functions, and factors arising out of socioenvironmental conditions. The differences in socioeconomic, nutritional status and physical activity level between the urban and rural population might influence postmenopausal women health more significantly than the hormonal changes. 

Menopausal status is accompanied by unfavorable levels of cardiovascular risk factors, like changes in body fat, distribution from gynoid to android pattern, abnormal plasma lipids, increased sympathetic tone, endothelial dysfunction, vascular inflammation, and increased blood pressure [2]. A major determinant of cardiovascular health and prognosis is the status of autonomic nervous system [3], although ignored by many clinicians. Abnormal autonomic control of heart rate is associated with an increased risk of cardiovascular morbidity and mortality [4]. The periodic fluctuations of heart rate are indicative of the relative contributions of sympathetic and parasympathetic components of autonomic nervous system to the heart and reduction of Heart Rate Variability (HRV) has been reported in several cardiac and noncardiac diseases.

Ethnicity, gender, and geography are powerful modifiers of community health. It is possible that geography is more powerful than any risk factor yet to be discovered. Geographic concentration of disease burden may have many causes including inadequate health care infrastructure, high level of poverty, and remote location [5]. Urban environment is associated with increased opportunities for mechanized or sedentary employment, consumption of processed foods, and other lifestyle characteristics associated with the development of cardiovascular diseases. A study also reported that rural to urban migration augments sedentary life style and undesirable eating habits among both men and women and thus accelerates the development of risk for diseases [6]. It has been shown that over the past fifty years prevalence of obesity, hypertension, hypercholesterolemia, and diabetes has increased significantly in urban and slowly in rural areas [7, 8]. Studies have shown that there is a greater incidence of cardiac disease among urban postmenopausal women due to the fact that they lead a sedentary lifestyle, while their rural counterparts are accustomed to greater amount of physical activity. However, another study comparing urban and rural women in United States for cardiovascular disease risk factors showed that the rural United States women had lower levels all types of HDL which is considered the protective cholesterol than that of urban United States women [9].

In the Indian scenario, the public health care system has typically concentrated on women of childbearing age, and once the women move out of this bracket they receive less attention unless they have access to private health care [3]. Menopausal health research also demands priority in Indian setting due to increase in life expectancy as a result of which women normally live between 10 and 20 percent of their lives in the postmenopausal state [10]. The United States Preventive Service Task Force has identified clear evidence that the health of women can be improved through preventive health screening [2].

This study attempted to evaluate HRV as risk factor for cardiovascular in postmenopausal women health contrasted between urban and rural women. The study also attempted to evaluate the association of indices of HRV with anthropometric measures among postmenopausal women. By categorizing postmenopausal women as high risk for cardiovascular diseases, findings of this study would play an important role in managing the disease, by early identification using cardiac autonomic function as a factor. The study hypothesized that rural postmenopausal women have higher heart rate variability, therefore lower degree of risk for cardiovascular disease as compared to urban women.

## 2. Materials and Methods

### 2.1. Subjects

A total of 60 postmenopausal women were recruited: 30 from urban and 30 from rural population based on location of their residence in South India. Subjects were recruited after taking a thorough clinical history. Women aged between 40 and 75 years, with absence of menstrual cycles for at least one year, were included. Subjects who had undergone hysterectomy, with history of chronic illness and on hormone replacement therapy, were excluded from the study. Informed written consent was obtained from all participants, and the experiment protocol was approved by Ethics committee of the institute. 

### 2.2. Experimental Protocol

All the participants reported for the study, after refraining from food, caffeinated or cocoa containing beverages for at least 2 hours. They were also instructed not to consume alcohol or tobacco 12 hours prior to recording. All the subjects underwent measurement of height, weight, and basal blood pressure. Height was measured to the nearest 0.1 cm without footwear, using vertically movable scale. Weight was measured to the nearest 100 grams using a digital scale. A basal recording of BP was done using Automatic Blood Pressure Monitor (Model SEM-2 Electronic Instrument, Omron Technologies). Subjects were also underwent the measurement of waist and hip circumstances. Waist circumferences were measured at the midpoint between the lower rib cage and iliac crest. Hip circumferences were measured at maximum circumference by placing the tape around the buttocks, without compressing the skin. Using the waist and hip circumferences the waist-hip ratio was calculated. 

HRV was assessed based on the RR intervals at resting condition. To quantify RR intervals, the analog ECG signal was recorded using lead II, to obtain a QRS complex of sufficient amplitude and stable baseline. ECG signals were conveyed through an A/D converter (after ECT Monitoring System, Niviqure Meditech Pvt. Ltd., Bangalore). The system has an integrated software which allows for recording of the ECG at a sampling frequency of 1024 Hz, with sampling rate of 8/s. 3 M standard chest electrodes (Ag-AgCl) were used. 4 electrodes were placed on the chest wall, one each on either side of the clavicle on the mid clavicular line and one each on the outermost and lowermost ribs.

ECG was recorded for a period of 5 minutes during which the subject was supine, awake resting, and normally breathing. Subjects were asked to avoid unnecessary movements during this period. The data so gathered was subjected to spectral analysis in the following way.

### 2.3. HRV Analysis

A noise-free electrocardiogram was obtained with the sampling frequency of 1024 Hz. Spectral analysis was performed off-line using Physionet. Data was edited manually for artifacts. HRV software uses a peak detection algorithm to find the “R” wave. The detection was done at a resampling rate of 4 Hz. Each detected “R” wave was considered as a data point. A minimum of 256 data points are required to perform a spectral analysis, for which a minimum duration of 5 minutes of ECG recording was obtained.

Spectral analysis is performed using a Fast Fourier Transform (FFT). The power is calculated in two bands. The 0.15-0.4 Hz band of RR power (high frequency) reflects parasympathetic nerve activity to the heart, while 0.04-0.15 Hz (low frequency band) is believed to reflect, at least in part, sympathetic nerve activity to the heart. In addition to absolute power, the data is also presented as normalized units and ratio of low frequency to high frequency (LF/HF) represents a measure of the balance of sympathetic and parasympathetic function.

### 2.4. Statistical Analysis

The analysis has been performed using SPSS 17.0 package. Data is expressed as mean ± SE. Values have been approximated to the third decimal. The total study population of 60 comprises of 2 independent groups with their respective data values. For the analysis of skewed data Mann-Whitney *U* test has been used. Linear associations between the different HRV indices and anthropometric measures were tested using Pearson's correlations. Differences were considered statistically significant at *P* < 0.05.

## 3. Results

**Figure 1:**
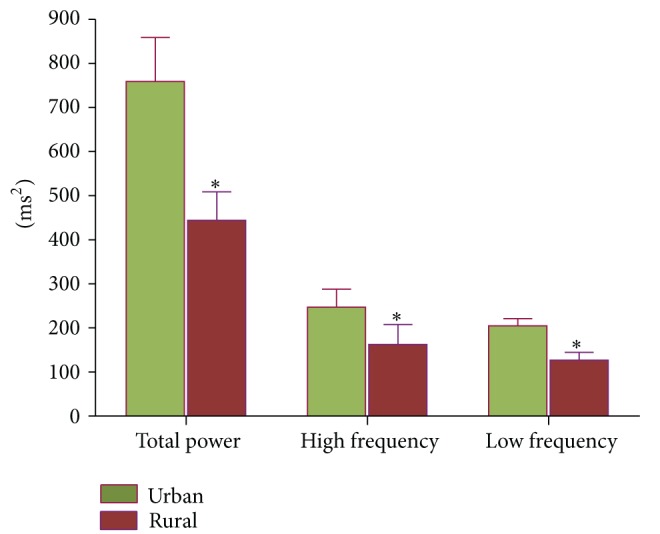
Comparison of HRV between urban and rural postmenopausal women. *Significant difference between the groups.

See Tables 1, 2, and 3 and Figure 1.

**Figure 2:**
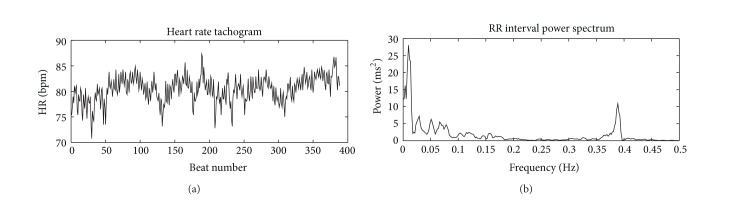
Heart Rate Variability Report 1. Heart rate tachogram and RR interval power spectrum from an urban subject. Greater area of the graph falls under high frequency band.

**Figure 3:**
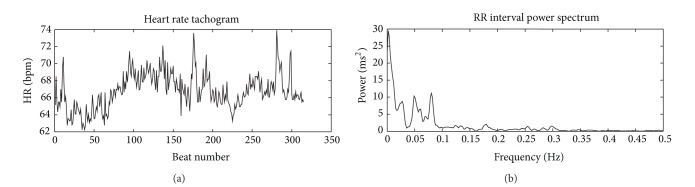
Heart Rate Variability Report 2. Heart rate tachogram and RR interval power spectrum from a rural subject. Lesser area of the graph falls under high frequency band as compared to Figure 2.

Figures 2 and 3 depict the format of result generated through Physionet.

## 4. Discussion

The present study hypothesized that the rural women would have lower risk for cardiac diseases since the idealized view of rural life is more active, least stressful, with healthy food habits, and greater social and community support. Therefore, the study compared the risk for cardio vascular disease by assessing the spectral analysis of Heart Rate Variability of thirty urban and rural postmenopausal women each.

Various techniques and maneuvers have been developed to detect the integrity of the sympathetic and parasympathetic nervous system. Most of the techniques such as cold pressor test, Valsalva maneuver, and the tilting table tests have focused on the evoked response of autonomic nervous system [11]. The evoked activities, however, might not reflect the tonic state of the body. Besides being a noninvasive study procedure, an important advantage of frequency-domain analysis of HRV is that it utilizes spontaneous fluctuation in heart rate to estimate tonic autonomic nervous functions which is sensitive index of cardiac morbidity. 

In this study, the participants of the two study groups were age-matched, and urban women were significantly overweight compared to the rural women. However, the waist circumference which is surrogate of abdominal obesity was significantly higher among rural women compared to that of urban women. The basal systolic, diastolic blood pressure, and resting heart rate were comparable between the study groups (Table 1).  Table 2 provides HRV indices among urban and rural women; the significant decreased total power of Heart Rate Variability among rural women indicates that they are associated with reduced overall cardiac autonomic modulation compared to that of urban postmenopausal women.  Table 3 provides a correlation matrix between HRV indices and anthropometric measures. The findings of the study show that absolute HF spectrum and LF spectrum significantly correlate with waist circumference, which is a surrogate of central obesity, but not with weight, BMI, and waist hip circumferences. 

Reduced HRV has been reported to be an independent risk factor for the development of coronary heart disease, cardiac sudden death, and all-cause mortality in women [12]. Therefore, the findings of this study indicate that rural postmenopausal women are associated with an additional risk beyond that of ageing and postmenopausal status when compared to urban women. Decreased high frequency component of the total variability in rural women observed, in this study, reflects the reduced overall protection of cardiac vagal modulation against cardiac mortality.

The observation of the present study is not in agreement with the study hypothesis. However, the findings of this study are comparable with the results of Broda et al. in which they reported that rural United States women had lower levels of all types of HDL which is considered to be the protective cholesterol, than that of urban women [9], which might explain to some extent our findings that rural women are at greater risk for CVD. Although studies reveal a 2-3-fold increase in the incidence of CHD among urban population than compared to rural population, they indicate a higher incidence of risk factors among the latter [13–15]. Chief among these are the increased calorie intake and greater percentage of saturated fat in diet consumed by the rural population, leading to increase in triglyceride to HDL cholesterol ratio, which has been described as an independent risk factor for CHD [13, 14]. Another factor which has not been taken into consideration in the present study is occupation. Our rural study population represents a region where majority of women are occupied with beedi rolling (hand rolled small cigarettes filled with tobacco) who handle about 35–40 kg of tobacco per month [16]. Studies have shown that a nearly 2-fold greater odds of CHD was found among women who were exposed to tobacco at work [17]. Significant correlation of HRV indices with waist circumference observed in this study is comparable with the report of Hagey and Warren, in which they report that the levels of central adiposity, which increase with age, contribute to the development of cardiovascular disease [18]. The causes underlying this geographical disparity in risk factors for cardiac disease among rural postmenopausal women could be inadequacy of healthcare infrastructure and the concomitant problems. A well designed and delivered rural health literacy program, addressing the health issues of elderly rural women, can motivate and bring in behavioral modifications necessary for good cardiovascular health. 

## 5. Conclusion

Indian rural postmenopausal women are associated with an additional risk for cardiovascular disease beyond that of ageing and postmenopausal status when compared to urban women. The causes for underlying this geographical disparity in HRV could be due to different central adiposities between the groups. Exercise intervention for postmenopausal women should aim to improve pattern of fat distribution that may reduce the risk for cardiovascular disease. 

## Figures and Tables

**Table 1:** Subject characteristics.

Parameter	Urban (*N* = 30)	Rural (*N* = 30)
Age (years)	54.79 ± 1.16	54 ± 1.15
Height (m)	1.574 ± 0.013	1.505 ± 0.009
Weight (kg)	63.133 ± 2.441	48.870 ± 1.538*
Body mass index (kg/m^2^)	25.4 ± 0.89	23 ± 0.79
Waist circumferences (cm)	76 ± 2.0	83 ± 2*
Waist Hip ratio	0.83 ± 0.01	0.85 ± 0.01
Basal SBP (mm of Hg)	138.83 ± 3.58	135.27 ± 3.65
Basal DBP (mm of Hg)	85.30 ± 1.55	82.80 ± 1.88
Resting HR (min^−1^)	73.300 ± 1.629	70.959 ± 1.888

Data expressed as mean ± SE.

*Significantly different across the two groups

SBP: systolic blood pressure; DBP: diastolic blood pressure; HR: heart rate.

**Table 2:** Comparison of HRV between urban and rural postmenopausal women.

Variables	Urban (*N* = 30)	Rural (*N* = 30)	*P* value
Total power (ms^2^)	759.207 ± 100.124	444.144 ± 65.608	0.000*
High frequency (absolute in ms^2^)	247.497 ± 41.739	163.308 ± 45.017	0.010*
Low frequency (absolute in ms^2^)	205.052 ± 26.133	127.340 ± 18.281	0.015*
High frequency (normalized)	53.172 ± 3.083	50.886 ± 3.984	0.897
Low frequency (normalized)	46.827 ± 3.083	49.114 ± 3.984	0.897
LF/HF	1.271 ± 0.278	1.624 ± 0.332	0.885

Data expressed as mean ± SE.

*Significantly different across the two groups.

**Table 3:** Correlation matrix between HRV indices and anthropometric measures.

Parameter	Total power	HF ab	LF ab	LF/HF
Weight (kg)	−0.122	−0.170	−0.135	−0.137
BMI (kg/m^2^)	−0.122	−0.176	−0.206	−0.202
Waist/Hip	−0.169	−0.116	−0.206	−0.108
Waist circumference	−0.226	−0.281*	−0.302**	−0.122

*Significant correlation.

**Highly significant correlation.
